# Are multi-cat homes more stressful? A critical review of the evidence associated with cat group size and wellbeing

**DOI:** 10.1177/1098612X211013741

**Published:** 2021-05-26

**Authors:** Lauren R Finka, Rachel Foreman-Worsley

**Affiliations:** Animal, Rural and Environmental Sciences, Nottingham Trent University, Brackenhurst Campus, Southwell, UK

**Keywords:** Household, conflict, welfare, health, *Felis silvestris*

## Abstract

**Objectives:**

The primary objective of this review was to conduct a systematic critical appraisal of published literature, in order to assess the evidence regarding the impact of cat group size on cat wellbeing in the domestic home. The secondary objectives were to: (i) identify additional social and environmental mediators of cat wellbeing in these contexts; and (ii) identify general limitations within the current evidence and provide recommendations for future studies.

**Methods:**

A systematic search of electronic databases (Scopus, Web of Science and Google Scholar) was conducted using targeted Boolean phrasing. Papers were retained for appraisal of full text where they included a comparison of both single (n = 1) and multi-cat (n ⩾2) domestic housing conditions and/or comparison of different multi-cat group sizes, within a single study, and where they compared outcome measures that were either behavioural and/or physiological and deemed as relevant indicators of cat wellbeing.

**Results:**

A total of 1334 unique papers were returned, 15 of which were retained. Of these papers, only four stated their primary aim to be an investigation of links between cat group size and cat wellbeing. Overall, the reviewed papers did not indicate consistent directions of effects regarding cat group size and outcome measures relevant to wellbeing. This was similar for the other social and environmental mediators identified.

**Conclusions and relevance:**

Inconsistency in results is likely due to the substantial methodological variation, limitations in measures used as indicators of wellbeing and limitations in general study designs and reporting. Results also highlight the complex, multifactorial relationships between cat wellbeing and various social and environmental factors. These may be as, if not more, important than absolute numbers of cats residing within a household. Due to the various limitations and general paucity of research, further studies are recommended to provide a suitable evidence base regarding impacts of multi-cat living on cat wellbeing in domestic environments.

## Introduction

As one of the most globally popular companion animals, the domestic cat experiences a diverse range of lifestyles and types of human management. Across these lifestyles, cats may encounter a range of environments and associated restrictions, from free living outdoors, to confinement within rehoming centres, or living within the domestic home. In each case, space and resource availability typically vary,^1–3^ as do the nature and degree of social interactions with both humans and conspecifics.^4–6^

At a species level, the domestic cat is capable of exhibiting an impressive level of social flexibility, enabling individuals to live in social groups with conspecifics and/or other species (including humans), or to alternatively live independent of social contact. At an individual level, some cats may transition across lifestyles and associated degrees of sociality within a generation, or even a single lifetime.^7^ For example, a singly housed pet cat may choose to stray from their domestic home and associate with other free-living cats in a colony. Equally, a solitary living cat born from feral or free-living parents may end up residing with humans and other cats within a domestic home. This may sometimes occur voluntarily on the cat’s part, although it is often the result of the cat being extracted from their original environment by humans. The capacity of individuals to adapt to these different lifestyles may depend on a complex interaction of factors. These include endogenous factors such as age, sex, neuter status and personality, and exogenous factors such as current resource availability and distribution, cat sex ratio, group size and familiarity and relatedness among conspecifics.^8–12^

In free-living contexts, domestic cats demonstrate diversity in both their spatial and social organisation, occupying a range of lifestyles from being primarily solitary^13^ to living in groups.^11^ When group living does occur, these are predominantly matrilineal, temporally stable and resource-dependent, forming around a clumped food source.^10^ For group members, familiarity and relatedness appear important mediators of affiliative interactions.^12^ Non-group members, especially when unrelated and female, are rarely tolerated and generally avoided.^11^ Much of the cat’s communicative repertoire is dedicated to the use of olfactory cues via semiochemicals, in combination with visual markers such as scratching.^14,15^ These behaviours facilitate remote forms of communication that avoid the need for close proximity to conspecifics or, importantly, non-group members. Indeed, distance-increasing strategies may be the preferred methods of avoiding inter-cat conflict in this species.^16^

Where cats are housed in confined spaces such as laboratories, rehoming centres or domestic homes, group living is usually determined and directly managed by humans. Group composition may therefore vary greatly compared with those formed by cats in free-living populations. For example, unfamiliar, unrelated cats of both sexes and from a range of backgrounds may be introduced and housed together as adults.^17,18^ Within these contexts, limitations of resource abundance and distribution relative to the requirements of group members may occur, with effective avoidance and distance-increasing strategies to reduce conflict less available. These vastly different circumstances to those of self-selecting populations may present challenges to group members that could be detrimental to their wellbeing.

In the rehoming centre context, studies assessing the stress levels of cats relative to their social housing type have produced mixed results. Some evidence suggests communal housing is associated with higher levels of stress,^19^ while other studies indicate higher stress in singly housed cats^20^ or no difference between housing types.^21^ However, a critical appraisal of the body of evidence identified several human, cat and environment factors that may be as, if not more, important than single or group housing alone.^22^ These factors included handling and husbandry styles,^20^ environmental disruption,^23^ socialisation history of cats towards humans and conspecifics^17^ and social stability of cat groups.^21^ While the authors pointed out that methodological limitations made direct comparison between housing types across studies difficult, these findings highlight the complex, multifactorial nature of social and environmental variables and their impacts on cat wellbeing.

In the domestic home, cats are frequently housed together.^24^ Here, variations in the nature of conspecific relationships are evident, but with agonistic encounters seemingly commonplace.^25,26^ Additionally, with a limited repertoire for proximal forms of conflict diffusion in confined environments,^27^ cats may utilise remote communicative strategies such as scratching and urine marking^14^ inside the home.^28^ These behaviours can be problematic for owners to manage successfully and may result in cat relinquishment.^29–31^ Multi-cat households may therefore be associated with negative welfare outcomes for cats, something routinely highlighted in the literature where advice for their management is discussed.^32–38^

Interestingly, the impact of single vs multi-cat living or variations in cat group size in the domestic home, and the role of potential mediating factors, does not appear to have been the primary research goal of many studies.^39^ Some useful information may be gleaned from published literature; however, relevant findings are typically a small component of the overall study,^2,25,40^ and thus not investigated or reported in detail. Given the global prevalence of multi-cat households (ranging from 41.7% of cat households surveyed in the UK^24^ to 73.6% in Italy^41^) and the seemingly common occurrence of inter-cat conflict in multi-cat homes,^25,26^ it is important to have an appropriate scientific evidence base to facilitate a better understanding of potential wellbeing impacts upon individuals and how these might be mitigated.

In this review, we therefore aimed to critically appraise the existing body of peer-reviewed literature, to provide a cohesive summary of current evidence on the relationships between cat group size (from single [n = 1] to multi-cat groups [n ⩾2]) and cat wellbeing in the domestic home, as measured by physiological and/or behavioural outcomes. Our secondary aims were to highlight specific risk factors associated with potential compromises to wellbeing in these contexts (such as various social and environmental parameters), as well as to highlight limitations within the current evidence base and provide recommendations for further research.

## Methods

### Focused clinical question

In cats kept in the home environment does cat group size result in differences in physiological and/or behavioural wellbeing?

### Literature search

A Boolean phrase was devised to search for relevant literature, based on our focused clinical question. As the authors were familiar with the research area, the phrase was optimised iteratively to ensure it returned all anticipated literature. The final phrase used was as follows:

(cat* OR feli*) AND (multi* OR singl* OR group* OR commun* OR discrete OR social* OR environment* OR hous* OR hom*) AND (welfare OR behav* OR enrich* OR stress* OR physi* OR problem* OR risk* OR conflict*)

Searches were completed in Scopus, Web of Science and Google Scholar in June 2020. These electronic databases were chosen due to the large quantity of literature they contained and their wide scope of source material. Searches were carried out on titles, keywords and abstracts and no date restrictions were imposed on returned literature.

From each database, the first 200 returns were exported into Mendeley. The next 200 titles were checked and exported if a potentially relevant paper was found. This continued until a consecutive batch of 200 papers with no apparent relevance to the review were returned. In total, 2200 papers were exported across the three databases – 1000 from Scopus, 800 from Web of Science and 400 from Google Scholar. These papers were collated in Mendeley and the ‘merge duplicates’ function used to ensure each paper was unique. After removing duplicates, 1334 individual papers remained for filtering.

### Filtering

For inclusion, both authors independently ensured the literature met the following criteria:

- A focus on domestic cats kept in the domestic home, including original observed or experimental data that was peer-reviewed, with the full text available in English.
- Comparisons across both single (n = 1) and multi-cat (n ⩾2) housing conditions and/or comparison of different multi-cat group sizes, within a single study, with outcome measures that were either behavioural, physiological or both, and were deemed as relevant indicators of (or at least likely to be highly associated with) wellbeing.
- Indicators of wellbeing included any outcome measures that provided potential information on the positive or negative welfare state of individuals, in line with modern concepts of animal welfare and overall quality of life.^42,43^ Papers where links between cat group size and (physical health) outcome measures were limited to the transmission of infectious disease were not included. However, we otherwise took a broad approach to the inclusion of wellbeing-linked measures (eg, human- and cat-directed aggression, house-soiling and urinary problems [ie, straining to urinate, vocalising when urinating, blood in urine, urethral obstruction], obesity, urinary and faecal cortisol concentrations, anxiety and owner accounts of ‘problematic’ or ‘concerning’ behaviours).

**Figure 1:**
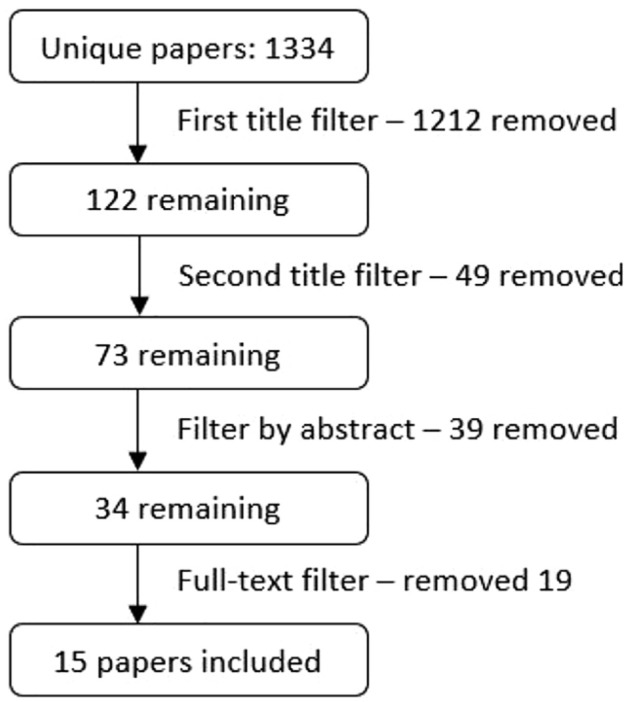
Stepwise filtering process of unique papers obtained through Scopus, Web of Science and Google Scholar database searches. Full-text filtering was completed by both authors who reached a consensus on the papers eligible for inclusion

Filtering was completed in a stepwise manner. Initially, titles were checked and papers not discussing domestic cats were removed. A second title review was completed to assess the potential relevance of the paper to the focused clinical question. Next, papers were filtered by abstract, and finally, a full-text review was completed. This process is illustrated in Figure 1. Filtering by title and abstract was completed by RF-W. Full-text reviews of all remaining literature were completed by both authors, with a consensus reached on all papers selected for inclusion, based on their relevance to the focused clinical question. For thoroughness, the references of all eligible papers were checked for their potential relevance for inclusion to ensure no papers had been missed. These checks yielded no additional papers.

### Data extraction and critical evaluation

Papers were divided at random between the authors for information extraction; this was undertaken using a standardised set of pre-agreed parameters that were considered relevant to the topic of the review and the focused clinical question. Pre-agreed parameters covered aspects such as cat and owner demographics, details of the cat’s living environment, social, environmental and wellbeing-linked variables measured, study intervention or comparisons and key findings relevant to the main study objectives (ie, significant relationships between cat wellbeing outcomes, multi-cat group size and other exogenous and endogenous factors). Extracted data were initially entered into a master table, which both authors then independently reviewed and jointly edited to ensure consensus of study interpretations and presentation of information. Once consensus of interpretations was confirmed, both authors jointly discussed the main limitations evident across the reviewed literature and categorised these into key themes. Limitations were identified on the basis of their impact on the strength of presented evidence in support of our focused clinical question. Data relevant to key findings were then exported into the tables presented within the results section, with the remaining information placed in a supplementary file (see Table S1 in the supplementary material).

## Results

From a total of 1334 unique studies initially identified, 15 were retained based on their relevance. These were taken forward for critical appraisal and data extraction (see Tables 1–3 and Table S1 in the supplementary material).

**Table 1:** Summary of the significant* reported links between cat group size (from n = 1 to n ⩾2) and various wellbeing outcomes identified across the 15 reviewed studies

Poorer wellbeing outcomes linked with greater numbers of cats in home	Wellbeing outcomes not linked with numbers of cats in home	Better or less poor wellbeing outcomes linked with greater numbers of cats in home
• Greater likelihood of cats exhibiting ‘behaviour problems’ and states of anxiety^2^ • Increased house-soiling and/or urinary problems (eg, straining to urinate, vocalising when urinating, blood in urine, urethral obstruction) frequency, or over-representation of house-soiling^25,28,46,52*^ • Increased conspecific aggression and conflict^36^	• ‘Behaviour problems’^45^ • House-soiling^44,47^ • Conspecific aggression^18^ • Obesity^47^ • Urinary cortisol to creatinine ratios^48^ • Faecal glucocorticoid metabolites^49,50^(for 50 this was only at the group level)	• Increased ‘quality of life’ scores and less ‘problem behaviour’^40^ • Fewer bites, aggressive behaviour and other negative interactions with humans^44,47,51^ • Lower faecal glucocorticoid metabolites^50^ (but only in cats aged <2 and when single cats were compared with groups of 3–4) • Increased conspecific affiliative behaviour^36^

* Reported links for one paper^52^ were purely descriptive

**Table 2:** Physical, social and individual cat characteristics significantly associated with more negative cat wellbeing outcomes

Factors relevant to the cat’s physical environment	Factors relevant to the cat’s social environment	Individual cat characteristics
• Cat having outdoor access,^18,28,45^ not having outdoor access,^28,51^ having a cat flap,^28^ having restricted outdoor access^2^ • House located in suburban area,^2^ house located in rural village^47^ • Less space per cat available (but not absolute home size),^2^ more inside space available (but not square metre per cat)^48^ • A ‘low’ number of litter trays provided (whether absolute or per cat unspecified), using crystal and recycled paper-type litter, trays in the same area, less frequent tray cleaning^46^ • Cat living in the house for >6 months^26^	• Owner living alone, being in a couple, being childless^2^ • Increased time cat left alone,^2^ fewer human–cat interactions^2,45^ • Owners having less cat knowledge^45^ • Higher number of humans in the household and more humans per square metre,^48^ socially active humans in the household,^40^ higher owner-reported human-social satisfaction^49^ • Owner under 55 years of age^47^ • Owner punishing the cat^45^ • First meetings of cohabiting cats described as unfriendly, fighting at initial introduction^18^ • New cat introduced to house within the past 6 months^26^ • Introduction of new cat to the household ‘did not go well’^26^ • Owner having degree level education^47^	• Cat being >2 years of age when in a multi-cat environment of 3–4 cats,^50^ being younger (1 year or 1–7 years)^26^ • Cat being 2–7 years or over 12 years^2^ • Cat being male,^25^ being female,^26,47^ being female and neutered,^2^ being female and intact^45,51^ • Cat being intact,^40^ being castrated^2^ • Cat being of mixed breed rather than pedigree,^47^ being a Persian breed^51^ • Cat being described as more ‘sedentary and shy’^26^ • Increased time cat living in the home^26^ • Cat being a stray, acquired from a shelter, from friends,^2^ being from a pet shop^51^ • Cat being declawed^45^ • Cat described as ‘tolerating’ rather than ‘liking’ or ‘disliking’ being petted by owner^50^ • Cat weighing 4 kg or more^2^ • Cat being acquired between 5 months and 1 year^2^

**Table 3:** Physical, social and individual cat characteristics significantly associated with better or less poor cat wellbeing outcomes

Factors relevant the cat’s physical environment	Factors relevant to the cat’s social environment	Individual cat characteristics
• Cat having free access to outdoors,^2,28^ or regular access (2–3 times a week) to outdoors^2^ • Cat having one litter box per cat plus one, having at least one food bowl per cat^26^	• More experienced cat owners^2^ • Owners handling their cats for several hours a day, and at consistent intervals throughout the day^2^ • New cat introduced to house within the past 6 months^26^ • Introduction of new cat to the household described as ‘went well’ by owner^26^	• Cat being young (<1 year or 1–7 years)^26^ • Cat being described as ‘active and curious’ by owner^26^ • Cat being described as ‘dependent on owner/clingy’ or having a ‘relaxed’ demeanor^28^

### General overview of studies

Nine studies were cross-sectional surveys (the most common study design) and incorporated analytical and/or descriptive elements.^2,18,25,26,28,40,44–46^ Of the six remaining studies, four were observational analytic cohorts (comprising one exclusively survey-based study^47^ and three including biological sampling^48–50^). The final two studies were retrospective and based on information gathered during behavioural consultations (one analytic^51^ and one purely descriptive^52^).

The following parameters varied both within and across study types:

i) Population sizes of both humans and cats. Survey-only studies ranged from 74 humans reporting on 74 cats^25^ to 12,010 owners reporting on 23,920 cats.^46^ Studies including biological sampling ranged from 12 owners sampling 18 cats^48^ to 60 owners sampling 120 cats.^50^

ii) Geographical regions sampled. Only one study included international respondents;^28^ these were predominantly from Brazil, the UK, Portugal, the USA and Australia. The remaining studies sampled within a single country, including the UK, Germany, Switzerland, Italy, Spain, Brazil, the USA and Australia. In several cases, sampling was limited to a specific region (eg, local vet clinics and regional newspapers^25,50^) or a single facility or organisation (eg, a university,^40,52^ shelter^18^ or veterinary clinic^51^).

iii) Demographic information collected, social and environmental parameters measured and general styles of information reporting. Details reported for both humans and cats varied but were generally brief. Human demographic information included the total numbers of individuals participating and their country of origin, with the exception of those^2,26,40,44,45^ where additional information such as the proportion of male/female respondents, their age ranges, average number of cats owned and ownership period were also mentioned. In general, slightly more demographic information was provided for cats including their ages, sex, breed, neuter and health status, whether declawed and source of origin. Again, these details varied across studies and in some cases were minimal.^25,44,46^ Collected measures relevant to the cat’s social and physical environment also varied in nature and detail across studies, from a broad range of measures^45,46^ to only a few.^25,40,49,51,52^ Measures included the absolute number of cats and humans per household and also per m^2^ within a household, neighbourhood cat density (known number of cats from other households in the immediate area), amount of human handling and time left alone each day, owner social behaviour and perceived quality of life, owner attachment to cat, cat ‘dominance rankings’, presence of other animals in home, basic resource provisions such as food, scratching posts, litter trays (in some cases their total amount per household and per cat, their location, cleanliness and substrate types), type of outdoor access, size of household, amount of indoor space available to the cat, and opportunities for climbing and play.

iv) Outcome measures associated with wellbeing. Outcome measures varied and mostly focused on negative (rather than positive) aspects of wellbeing. ‘Problem behaviour’ was one of the most commonly assessed variables and was mainly used as an umbrella term to represent behaviours considered problematic or concerning to owners (eg, anxiety, scratching furniture, aggression [conspecific and human directed], house-soiling, undesirable sexual behaviour, liveliness, destructiveness, vocalisation, escaping, roaming and hunting), although examples of reported ‘problem behaviours’ varied slightly across studies.^2,45,51,52^ Several papers focused on specific behaviours that might otherwise have been included under the generic term of ‘problem behaviour’. These behaviours included house-soiling and urinary issues (eg, straining to urinate, vocalising when urinating, blood in urine, urethral obstruction^25,28,46,52^), the owner’s perception of the cat’s general behaviour (eg, level of anxiety, timidity, nervousness and shyness^2,26,28,44^), as well as human-directed aggression^44,47,51^ and inter-cat conflict.^18,44,47,51^ In some cases, physical or physiological indicators such as cat obesity,^2^ faecal glucocorticoid metabolites^49,50^ and urinary cortisol:creatinine ratios^48^ were sampled, although most studies relied solely on owner reports of cat health and wellbeing based on behavioural outputs. These ranged from structured, quantitative observations (eg, number and location of urine marks in the home over a 2-week period,^25^ frequency of cat fights per week^18^ and number of cat bites within the past year^47^), to general impressions of the cat such as their demeanour.^26,28,50^ In total, only three studies included biological measures relevant to wellbeing (eg, faecal glucocorticoid metabolites, urinary cortisol:creatinine ratios^48–50^).

v) Analytical approaches. A range of analytical approaches and subsequent tests were applied to a suite of demographic and cat management variables. Variables were tested relative to few,^2,45^ and greater^47^ amounts of wellbeing-related outcomes. For example, in Heidenberger (1997)^2^ cat/owner/housing variables were assessed individually for their relationship with the presence of cat problem behaviour (ie, yes/no) and anxiety (present/absent). In Roberts et al (2020),^47^ cat/owner/housingvariables were tested for their relationship with cat obesity, periuria, cat bites to owner and other negative cat–owner interactions, as well as agonistic and non-agonistic interactions with conspecifics.

Group sizes within ‘multi-cat’ groups that were compared with singly housed cats varied both within and across studies. For example, in one study, outcome measures for singly housed cats were compared with those from cats housed in pairs.^2^ In another, measures for singly housed cats were compared with those from cats housed in multi-cat groups that ranged from two up to 30 cats per ‘multi-cat’ group.^47^

### Key findings

Overall, across the reviewed papers, results did not indicate consistent directions of relationships between numbers of cats within a household and outcome measures relevant to cat wellbeing. Four of the papers included mixed results (ie, increases in cat group size were linked to both positive and negative wellbeing outcomes), depending on the specific outcome in question^36,44,47^ or the variables outcomes were being tested against.^50^ In total, six of the 15 papers provided evidence indicating greater numbers of cats within the home were significantly associated with poorer wellbeing outcomes and six papers provided evidence indicating the opposite trend. A total of seven papers also provided evidence indicating no links between the number of cats in the household and wellbeing outcomes (see Table 1).

### Additional social and environmental mediators of cat wellbeing

A range of other variables (summarised in Tables 2 and 3) were reported as being significantly linked to the wellbeing outcomes measured. These included exogenous factors covering aspects of the cats’ physical (eg, outdoor access, indoor space available, litter tray provisions) and social (eg, time alone, human density and level of human social activity) environment, in addition to various endogenous factors (eg, breed, sex, age, neuter status). Across the literature, a range of non-significant relationships between wellbeing outcomes and physical, social and cat characteristics were also identified, although these were too many to enable their concise reporting within this review (and were also considered to be largely outside its scope).

### Key limitations

As in Finka et al (2014),^22^ the substantial variation in study methodologies and reporting styles across the literature made direct between-study comparisons problematic. Among the reviewed studies, a series of key limitations were identified, which could typically be assigned to one of two categories; limitations relating to i) the general scientific quality of the study design, analysis and reporting and ii) the relevance of the study to our focused clinical question. Collectively, these limitations restricted the strength of available evidence and thus the overall conclusions that could be drawn regarding relationships between cat group size and cat wellbeing.

### ii) General scientific quality

#### Owner and cat sampling bias

Owners were typically self-selecting, with recruitment methods involving advertisements within veterinary centres and universities, or via online survey sharing.^2,25,26,28,40,44,45–50^ Self-selection sampling, specifically through online surveys, is often associated with responder bias, with some subgroups tending to be over engaged (ie, women) and others under engaged (ie, the elderly or those from lower socioeconomic backgrounds^53^). Of the five studies reporting responder gender,^2,26,40,44,45^ all indicated higher proportions of owners identifying as female, with percentages ranging from 60.3%^45^ to 96%.^44^ No studies reported details of age distributions or socioeconomic status; therefore, other responder biases may be present but unaccounted.

Many studies utilised demographic data and/or wellbeing measures from a single^18,25,^28^,40,45,47,51,52^ or limited^49^ number of cats within each multi-cat household, as opposed to sampling all members. In some instances,^28,49,40^ studies requested the owner select a focal cat from their multi-cat household to report on. Such methods may have unwittingly introduced cat sampling bias. For example, owners may have selected the cat that they were most attached to, causing more positive reporting due to ‘pet enhancement’ effects.^54^ Equally, owners might have selected the cat at the extreme ends of a behaviour spectrum, such as individuals exhibiting few or many ‘problematic behaviours’, or those involved in a lot, or minimal, inter-cat conflict. This method of sampling therefore cannot account for potentially important variations in behaviour and wellbeing parameters within each multi-cat group, which may be a particularly pertinent issue where owner bias occurs in focal cat selection.

#### Analytical approach

Survey-based papers typically tested large amounts of explanatory variables without specific a priori rationale provided. Through multiple statistical comparisons, five studies recognised the possibility of introducing type 1 errors,^18,26,28,44,45^ with four subsequently adjusting their significance thresholds, primarily through Bonferroni corrections.^18,26,44^ One study did not adjust the significance threshold as they posited that type 2 errors were more cause for concern than type 1, based on their study design.^28^ Small sample sizes were also recognised as a concern resulting in possible type 1^47^ or type 2^48^ errors occurring. One study did not include any statistical analysis of their data.^52^

For several studies, it was unclear which variables were included in the various analyses or what the response and explanatory variables were.^2,44,46,51^ Subsequently, it was unclear whether certain variables had simply not been considered in the analyses performed, or whether they had, but the results were omitted due to their non-significance. In some cases, this was unclear in the main text although further details and test outputs were included within appendices or supplementary material.^28,47^

### ii) Study relevance to focused clinical question

#### Stated aim not specifically focused on the impacts of multi-cat living on cat wellbeing

Most papers did not have a specific focus on how multi-cat environments may affect cat wellbeing.^2,18,25,26,28,40,44–46,51,52^ Typically, the number of cats within a household was one of many variables considered when exploring living conditions and cat management. The focus of these papers ranged from investigating factors associated with reported ‘behavioural problems’ (primarily house-soiling or ‘urinary problems’; eg, straining to urinate, vocalising when urinating, blood in urine, urethral obstruction^25,28,52^), to exploration of cat caretaking and management practices.^2,40,45,46^ Consequently, only small portions of the analyses and subsequent results from each paper were relevant to our focused clinical question.

#### Limited validity of outcome measures as indicators of wellbeing

All three studies that collected physiological data focused exclusively on excreted cortisol values, measured from faeces^49,50^ or urine.^48^ While objective, such measures are potentially limited in their ability to provide information on the overall wellbeing of individuals due to their lack of specificity concerning emotional valence.^55^ This limitation is particularly pertinent where parameters are not interpreted in combination with other behavioural indicators of wellbeing,^56–58^ such as sickness and stress-linked behaviours or physical health indicators. Other endogenous factors not directly associated with wellbeing such as age, sex and neuter status may all potentially influence cortisol levels,^59^ and should therefore be suitably controlled for within study designs or analyses.

For the remaining papers, wellbeing-related outcomes were predominantly based on the subjective reports of owners such as perceived cat ‘problem behaviours’,^2,18,25,26,28,40,44–47,51,52^ how timid/confident^44^ timid/easy going^50^ and anxious^2,45^ the cat was, the quality of the cat–human relationship^50^ and accounts of conspecific and human-directed affiliative and agonistic behaviours.^18,26,40,44,47,51^ These were often based on owner observations or recollections of cats’ behaviour over unspecified time periods.^2,28,40,44,45,47,51,52^ While cat ‘problem behaviour’ was one of the most commonly sampled outcome variables,^2,26,40,45,51,52^ this measure was mostly presented anthropocentrically, rather than being specific to cat wellbeing. As such, this measure might reflect behaviours that may or may not represent compromises to cat welfare. For example, behaviours such as furniture scratching, liveliness, destructiveness, vocalisation, escaping, roaming and hunting may be problematic for owners^2^ but simply part of the cat’s natural behavioural repertoire.^60^ Other behaviours such as spraying and house-soiling might indicate problems with management or care provision, although their presence may not be directly correlated with relative wellbeing.^61^

#### Variation in ‘multi-cat’ groups and methods of comparison

There was substantial variability in the type of information provided on the total number of cats within each multi-cat group and a general lack of specificity over total numbers. Multi-cat groups were typically treated as categorical variables, ranging from pairs, ‘groups of three or four’, ‘three or more’ or from two to 30 individuals, depending on the study.^18,26,44,45,47,48,50,52^ Only one study provided the exact number of cats within each household sampled.^49^ Five studies provided the mean number of cats per household, with or without the standard deviation,^2,45–48^ two additionally provided the range across their multi-cat households^45,47^ and one the median and interquartile range.^47^ Four studies provided no information regarding the quantity of cats within the multi-cat households sampled.^25,28,40,51^

Analysis of multi-cat groups also varied between studies. Six papers appeared to treat single and multi-cat households as binomial variables despite likely or confirmed variation within the number of cats within each separate multi-cat home.^25,28,46,47,49,51^ Five studies split multi-cat households into discrete categories; for example, pairs, groups of ‘three or four’, groups of ‘three or more’^2,^44^,45,48,50^ for analysis between groups. However, these studies often excluded multi-cat households of certain sizes; one study excluded pair households and compared single cats with groups of three or four,^48^ one paper excluded households of four cats or more^18^ and two papers excluded households of five cats or more.^26,48^ Three studies contained minimal to no statistical analysis between single and multi-cat households or multi-cat households of different sizes.^6,18,52^ Additionally, in two studies it was unclear how such variables were treated within the statistical analysis (ie, binomial, discrete categories, or if the specific numbers of cats in each house were treated as continuous variables^2,40^).

## General discussion

A total of 15 papers were critically reviewed to establish the current evidence base for links between cat group size (eg, from single [n = 1] to multi-cat groups [n >2]) and wellbeing within the domestic home. Our appraisal indicated that in most cases, differences in the number of cats within households were significantly linked to various wellbeing outcomes. However, similar to Finka et al (2014),^22^ the direction of these effects was inconsistent, and in some cases apparently contradictory (eg, larger group sizes were associated with more^2^ but also less^40^ ‘problem behaviour’). This is perhaps unsurprising given the diverse methodological approaches used, which resulted in large variations in sample sizes, population demographics, variables measured and types of analyses performed, as well as the style and detail of general reporting. In particular, the lack of specificity of, or variations in, the size of cat groups being compared, as well as the diversity of outcome measures sampled and their limitations as indicators of wellbeing, made between-study comparisons difficult. Thus, while findings from various studies may appear contradictory,^2,40^ the details included within their methodological and statistical reporting meant it was not possible to make anything other than surface-level comparisons.

For most papers, assessing the impact of cat group size on wellbeing was not the primary aim of the study. Thus, where significant links were identified, these were often a result of multiple testing between variables and wellbeing outcomes, in most cases with limited biological rationale or justification provided. Where methodological reporting made it unclear which variables had been tested,^2,44,46,51^ it is reasonable to assume only significant results were reported, given the systemic bias towards significance reporting across scientific disciplines.^62–64^ While type 1 errors associated with multiple testing may be avoided by performing Bonferroni corrections,^18,26,44^ this may in turn increase the probability of type 2 errors, particularly in studies using small sample sizes.^28,65^ Therefore, providing a clear rationale for all tests conducted, combined with clear reporting of effect sizes and *P* values for each, may be preferable to performing power-reducing corrections and selective result reporting.^65^ Collectively, the limited cross-study comparability, inconsistency in the direction of relationships identified, potential omission of non-significant (but relevant) results, combined with the likelihood of both type 1 and type 2 errors, all serve to limit the strength of relevant evidence and thus our current understanding of this topic.

While we highlight the limited scope of the wellbeing-linked measures sampled and their reliability and validity as indicators of cat welfare, we acknowledge that assessing cat wellbeing was not the primary aim of most papers. Therefore, our criticisms relating to measure quality are more to highlight important considerations for future research. These should ideally take a triangulated approach to wellbeing assessment^55,66^ and avoid the reliance on single measures in order to infer welfare.^48,50^ Cat wellbeing may be optimally investigated by incorporating a range of both subjective and objective measures, across physical, behavioural and biological parameters, utilising validated tools where they exist.^67,68^ Measures should also be considered relative to their ability to capture welfare compromise or stress levels across suitable time periods. For example, cortisol concentrations taken from hair samples might give an indication of blood cortisol responses over a longer period than faeces or urine, although each method of cortisol sampling comes with various limitations.^55^ An absence of negative wellbeing outcomes may not necessarily indicate an optimum welfare state or good quality of life.^42^ The presence of behaviours or indicators associated with positive anticipation, play, affiliative social behaviour, relaxation and contentment should therefore also be included^43,69^ to provide a more holistic view of individual wellbeing.

As in Finka et al (2014),^22^ a range of social, environmental and cat-specific factors outside of cat group size were found to be significantly linked to the wellbeing outcomes of interest (see Tables 2 and 3), suggesting these complex, multifactorial relationships extend beyond free-living and rescue contexts and into the domestic home. However, differences in the variables collected across studies, as well as their direction of effects, made it difficult to form firm conclusions on specific risk factors for cat wellbeing in these contexts. Furthermore, the stability and generalisability of most findings to broader populations of cats and their owners is unclear, given the presence of sampling biases (eg, gender skew, participant and cat selection, sampling limited to specific geographic regions).

What these results do highlight is the importance of considering a range of variables as potential confounds or covariates when investigating links between cat group size and wellbeing in the domestic home. Based on our current understanding of observations from both free-living and confined environments, these should consider available space per cat,^70,71^ and resource availability and distribution,^72^ as well as the composition and characteristics of multi-cat groups. For cat characteristics and group compositions, their sex and sex ratios,^73,74^ socialisation history with conspecifics,^75^ relatedness and familiarity,^6,12,73^ as well as the nature of conspecific relationships (ie, generally affiliative, agonistic, tolerant or avoidant) and individual personality are potentially all important.

It is also worth noting that across studies, humans behaviour, both that directed towards the cat and towards other humans, was frequently linked to wellbeing outcomes. For example, cats receiving fewer interactions with humans^45^ and being left alone for longer periods^2^ were associated with more reported ‘behaviour problems’ and anxiety, respectively. However, in other studies, increased human presence in the home and higher levels of human social activity were associated with higher urinary^48^ and faecal cortisol^49^ concentrations. Additionally, higher faecal cortisol concentrations were identified among cats described as ‘tolerating’ rather than ‘liking’ or ‘disliking’ being petted by their owners.^50^ However, as previously highlighted,^55^ such physiological measures should be interpreted with caution, especially when considered in isolation from relevant behavioural indicators. While scientific investigations into the impacts of the human-social environment upon cat wellbeing and their underpinning mechanisms are still in their infancy,^40,76,77^ it is likely that they may also act as important mitigators of cat wellbeing in the domestic home.

## Conclusions and recommendations for future research

The current body of evidence did not indicate consistent directions of effects regarding cat group size and outcome measures relevant to wellbeing. These results highlight the potentially complex, multifactorial relationships between cat wellbeing and various social and environmental factors. Such factors may be as, if not more, important to consider than simply the number of cats residing together within a household.

However, given the paucity of current literature investigating the impact of group living on the wellbeing of cats within the domestic home, further research is required to provide a larger, better quality, evidence base. While several studies produced seemingly contradictory findings, it is possible that these are a function of the substantial variation in methodological approaches used as well as the cat owner populations and wellbeing-linked measures sampled.

While cross-sectional survey designs (the most commonly used method within this review; see Table S1 in the supplementary material) potentially offer a practical way to sample large international populations, they are limited in their ability to identify causality among variables of interest and are notorious for sampling biases.^53^ Considering the nature of the research topic (eg, cats residing in the homes of private citizens), randomised controlled trials are unlikely to be feasible for future exploration of the relationship between cat wellbeing and group size in these contexts. Large (ideally matched) cohort studies comprising populations with demographic features that support generalisability of findings may therefore be the next best option in terms of evidence quality.^78^

Such studies should aim to collect suitably valid measures of cat wellbeing. However, we acknowledge that this is not without its challenges, given the limitations associated with physiological measures, subjective owner reports of cat behaviour and the need for practical measures. We would also suggest the collection of other potentially important social and environmental parameters (see above). Such information could be used to provide sufficient demographic context regarding study populations to support effective cross study comparison, or ideally be included as possible covariates or random effects along with the main explanatory variable (eg, group size) within statistical analyses. Treating numbers of individuals within each household sampled as continuous rather than categorical variables, or at least more balanced group sizes within discrete categories, will likely provide a more sensitive measure of cat group variation and potential links to cat wellbeing. Lastly, it is recommended that studies provide clear rationale for the inclusion and subsequent testing of all response and explanatory variables, as well as the full reporting of all test statistics, even when not significant.

## Supplemental Material

Table S1 Click here for additional data file. Data relevant to key findings
